# 580 Intravascular Targeted Temperature Management Line to Treat Hypothermia in a Major Burn Patient: Case Report

**DOI:** 10.1093/jbcr/iraf019.209

**Published:** 2025-04-01

**Authors:** Joan Dolinak, Emma Steinmetz, Jean Luc Francois

**Affiliations:** Upstate University Hospital; Upstate University Hospital; Upstate University Hospital

## Abstract

**Introduction:**

Intravascular Targeted Temperature Management (TTM) cooling techniques have been well-established in post-ROSC patients to preserve neurologic function, however, there is less literature available discussing use of these systems to warm hypothermic patients. There are case reports and small scale studies describing patients benefiting from targeted intravascular rewarming in the setting of trauma and significant hemorrhage with good results.

TTM appears applicable in the burn patients given their susceptibility to hypothermia and its associated negative impact on mortality, coagulation, immunity and cardiac arrhythmias. Perioperative hypothermia remains a major issue in management of burn patients despite routine implementation of active and passive rewarming strategies.

The use of intravascular thermoregulatory catheters could safely address this issue inherent to our patient population.

**Methods:**

We present the case of a patient who sustained a major burn and required intravascular warming for refractory hypothermia that would have delayed operative management.

The adult patient sustained >50% TBSA, was decontaminated on scene, then transported to the regional burn center ER. Temperature on arrival 34.4 C. Patient was transferred to the treatment room for rewarming, cleansing and escharotomies. Maximum core temperature via foley catheter monitor was 37.5 C. Following application of full strength Dakin’s soaks, core temperature decreased to 35.3 C. Despite continuation of all active and passive warming techniques of a hot room, 3 heated IV lines, heated ventilator, underlay warmer, Bair hugger and heated dialysis, temperature only increased by 0.3 degrees over three hours. A TTM central line was placed in his left internal jugular, and in addition to the aforementioned methods, was used to rewarm the patient to 37.5 C to meet eligibility for operative management.

**Results:**

Following TTM initiation, core temperature increased by 0.2, 0.8 and 0.7 degrees Celsius in the 1st, 2nd and 3rd hours respectively. The patient became eligible for initial tangential excision within 24 hours of arrival and remained normothermic in the OR with continuation of active and passive rewarming strategies. The patient had no thrombotic events during the hospitalization.

**Conclusions:**

This patient likely benefited from the short term use of an intravascular TTM line to rewarm his core temperature to allow an early major excision. A review of literature suggests this may be an underutilized option for burns and potentially help in the OR to help manage the core temperature of patients undergoing major excisions. Additional study of intravascular temperature management in the burn population could prove to be a successful adjunct for care.

**Applicability of Research to Practice:**

Description of case where use of an intravascular TTM line reduced time of correction of hypothermia and allowed the patient access to the OR for a safer early excision.

**Funding for the Study:**

N/A

